# Modulated self-reversed magnetic hysteresis in iron oxides

**DOI:** 10.1038/srep42312

**Published:** 2017-02-21

**Authors:** Ji Ma, Kezheng Chen

**Affiliations:** 1 Lab of Functional and Biomedical Nanomaterials, College of Materials Science and Engineering, Qingdao University of Science and Technology, Qingdao 266042, China

## Abstract

The steadfast rule of a ferromagnetic hysteresis loop claims its saturation positioned within the first and third quadrants, whereas its saturation positioned in the second and fourth quadrants (named as self-reversed magnetic hysteresis) is usually taken as an experimental artifact and is always intentionally ignored. In this report, a new insight in this unique hysteresis phenomenon and its modulation were discussed in depth. Different iron oxides (magnetite, maghemite and hematite) with varying dimensions were soaked in FeCl_3_ aqueous solution and absorbed Fe^3+^ cations due to their negative enough surface zeta potentials. These iron oxides@Fe^3+^ core-shell products exhibit well pronounced self-reversed magnetic hysteresis which concurrently have typical diamagnetic characteristics and essential ferromagnetic features. The presence of pre-magnetized Fe^3+^ shell and its negatively magnetic exchange coupling with post-magnetized iron-oxide core is the root cause for the observed phenomena. More strikingly, this self-reversed magnetic hysteresis can be readily modulated by changing the core size or by simply controlling Fe^3+^ concentration in aqueous solution. It is anticipated that this work will shed new light on the development of spintronics, magnetic recording and other magnetically-relevant fields.

Self-reversed magnetic hysteresis (SRMH) refers to a unique hysteresis phenomenon with antiparallel magnetization to an applied magnetic field and hence leading to an inverted hysteresis loop with “negative” net area. It has typical diamagnetic features, yet it shapes like ferromagnetic loops. It has often been taken that the “negative” area of the hysteresis loop violates the thermo-mechanical second law, so that SRMH is seen as an experimental artifact probably arising from inappropriate or asymmetric sample positioning and the misinterpretation of experimental data^1^. However, a bold prediction of SRMH was firstly made by Néel in 1951^2^ and in the same year an astonishing discovery of SRMH in volcanic rocks from Haruna was firstly reported by Nagata and his coworkers^3^. From then on, there is mounting evidence that SRMH can be readily observed in some minerals with the presence of two magnetic phases^4^,^5^,^6^,^7^. The possible mechanism of SRMH can be described by experimental evidence that the preformed metastable “*x* phase” becomes weakly and normally magnetized in the presence of an applied magnetic field during cooling process. This “*x* phase” is negatively exchange-coupled with a subsequently-formed stable phase at a lower temperature. The strongly magnetized stable phase together with its negative exchange coupling to the “*x* phase” is the root cause of SRMH.

SRMH phenomenon is of fundamental importance in academia owing to its interdisciplinary feature. Based on careful investigations of myriad rocks all over the world, geologists have found thermoremanent-magnetization direction of some rocks or minerals is totally opposite to the present geomagnetic direction, and hence they believe the Earth’s magnetic field has reversed its polarity many times throughout the earth history. Such discredit geological speculations may well misconstrue the reality if the SRMH phenomenon is really relevant^8^. Besides, the severely contentious views of “to be or not to be” as for SRMH inhibit its tremendous potential applications in spintronics and handicap its further applications in other fields. Despite the “discovery” progress achieved to date, a major hurdle to a more definitive understanding of the intrinsic SRMH mechanism is to develop a simple and effective method to synthesize a high yield of man-made materials with tunable SRMH features. In this report, we propose a facile and versatile method to scalable synthesis of SRMH iron oxides. By studying the phenomenological results in depth, we find an effectual and versatile approach to tailoring the desired SRMH character of the as-synthesized materials. Zero-dimensional (0-D) magnetite/maghemite (Fe_3_O_4_/γ-Fe_2_O_3_) nanoparticles (NPs) is firstly chosen as a canonical example to validate the very existence of SRMH and explore its modulation way. Then the two-dimensional (2-D) pine-like and three-dimensional (3-D) flower-like α-Fe_2_O_3_ architectures further confirm the applicability and effectiveness of this way to yield tailorable SRMH behaviors.

## Results and Discussion

**Figure 1:**
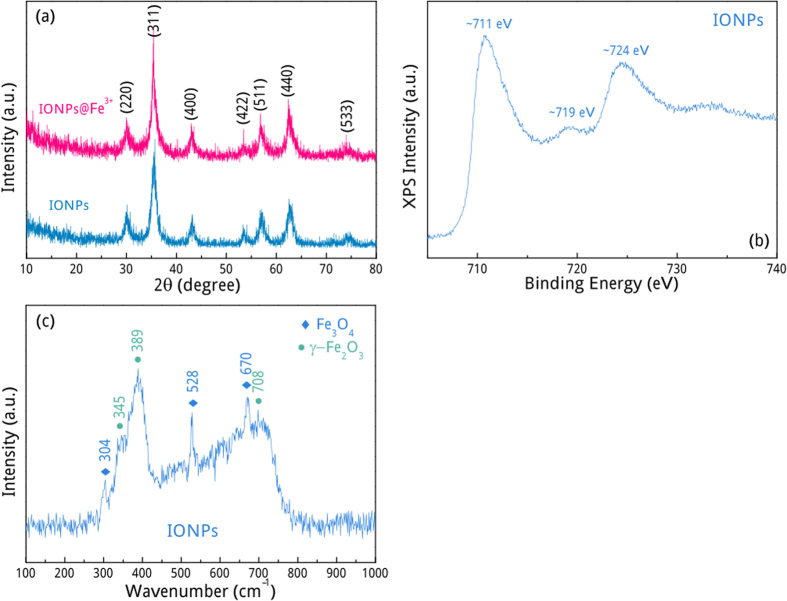
(**a**) XRD patterns, (**b**) XPS spectrum, and (**c**) Raman spectrum of the as-synthesized products.

The chemical composition of the as-synthesized products was firstly investigated by XRD measurements. Figure 1a shows these iron oxide nanoparticles (IONPs) and IONPs@Fe^3+^ products may contain Fe_3_O_4_ as a main phase owing to the reducing environment created by the ascorbic acid reagent. On account of the detection limit (~5 wt.%) for XRD measurements, XPS spectrum of bare IONPs was measured to further detect the possible existence of other iron oxides. Figure 1b gives the binding energy of Fe 2p, in which two distinct peaks at ~711 and ~724 eV correspond to Fe2p_3/2_ and Fe2p_1/2_ levels, respectively^9^. The presence of a satellite peak around 719 eV further demonstrates that IONPs may also contain γ-Fe_2_O_3_ phase^10^. A Raman spectrum measurement was then performed to validate the real presence of γ-Fe_2_O_3_ phase. Figure 1c demonstrates that two T_2g_ modes (Fe-O asym. bend) 304 and 528 cm^−1^, and one A_1g_ mode (Fe-O sym. str) 670 cm^−1^ are the characteristic features in Fe_3_O_4_, while the bands at 345, 389 and 708 cm^−1^ are derived from E_g_ (Fe-O sym. str), T_2g_ (Fe-O asym. bend) and A_1g_ (Fe-O sym. str) modes in γ-Fe_2_O_3_, respectively^11^. No other impurity phases are detected. After cation adsorption, the amount of surface Fe^3+^ cations was determined by ICP measurements. Considering cation adsorption can be suppressed by increasing the ionic strength, these IONPs@Fe^3+^ samples were separately soaked into supersaturated NaCl aqueous solutions for 24 h. After centrifugation, the Fe^3+^ concentration of supernatants were determined to be 66, 62, 59 and 55 mg/g for S1, S2, S3, and S4 by ICP measurements, respectively. These parameters are summarized in Table 1.

**Figure 2:**
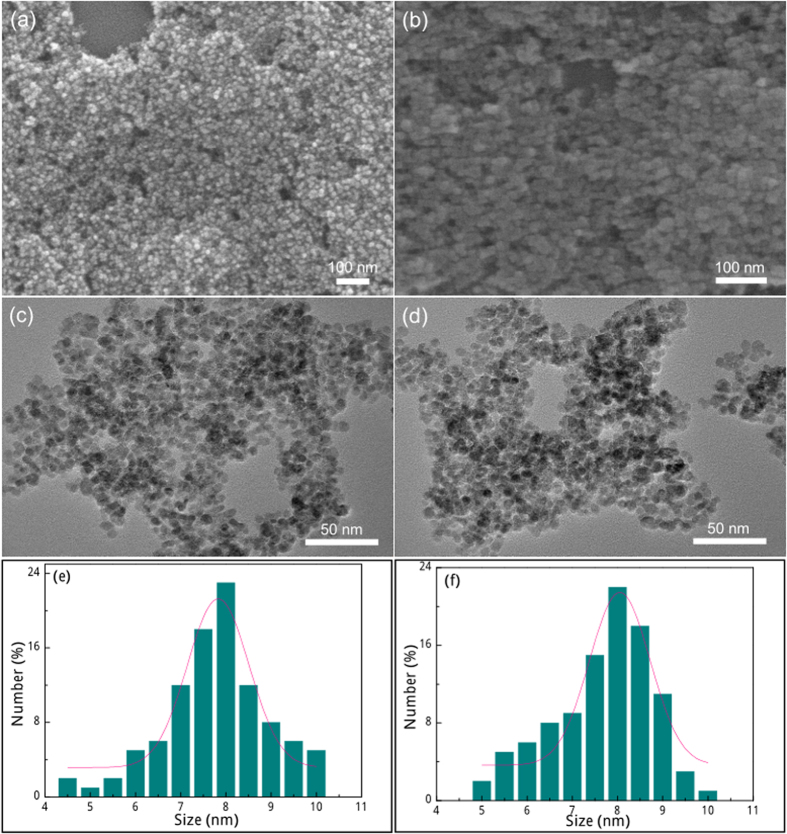
(**a,b**) SEM and (**c,d**) TEM images of the as-synthesized IONPs (panel a and c) and IONPs@Fe^3+^ (panel **b** and **d**) products. Statistical particle size distribution for (**e**) IONPs and (**f**) IONPs@Fe^3+^ products.

Electron microscopes were used to observe the morphology and size distribution of the as-synthesized products. These bare IONPs and IONPs@Fe^3+^ are of 0-D spheroidal morphological features (Fig. 2a and b), and their average particle sizes can be estimated by statistically counting a hundred isolated particles in Fig. 2c and d, respectively. The diameters of bare IONPs distribute around a peak value of 8 nm and the width of the distribution at half-maximum being 1.8 nm (Fig. 2e). After Fe^3+^ cation adsorption, IONPs@Fe^3+^ distributes in size in the range of 5–10 nm (Fig. 2f), and also has a peak value of 8 nm.

The successful synthesis of IONPs@Fe^3+^ is mainly ascribed to the negative enough surface zeta potential of bare IONPs (ca. −22.1 mV) and their strong electrostatic attraction to the positively charged Fe^3+^ cations in FeCl_3_ aqueous solution. This synthetic route is quite facile, highly repeatable, easily controllable and commonly versatile. Numerous IONPs@Fe^3+^ with different surface zeta potentials can be readily obtained by intentionally varying Fe^3+^ concentration in the FeCl_3_ aqueous solution. In this work, four sets of FeCl_3_ aqueous solution with Fe^3+^ concentration of 5, 2, 1 and 0.5 mol/L were utilized as the absorbing solution, and the obtained IONPs@Fe^3+^ products are marked as S1, S2, S3 and S4, respectively. Their zeta potential values are measured to be 19.3 mV (S1), 15.8 mV (S2), 2.7 mV (S3) and −9.2 mV (S4), which decrease with lowering Fe^3+^ concentration in FeCl_3_ aqueous solution (Table 1).

**Figure 3:**
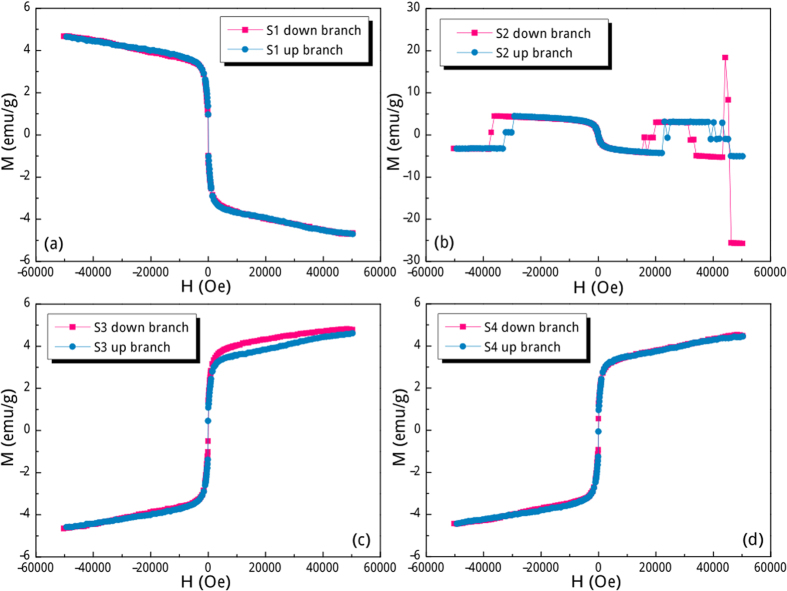
Room-temperature magnetic hysteresis loops of IONPs@Fe^3+^ with different zeta potentials: (**a**) 19.3, (**b**) 15.8, (**c**) 2.7 and (**d**) −9.2 mV.

**Figure 4:**
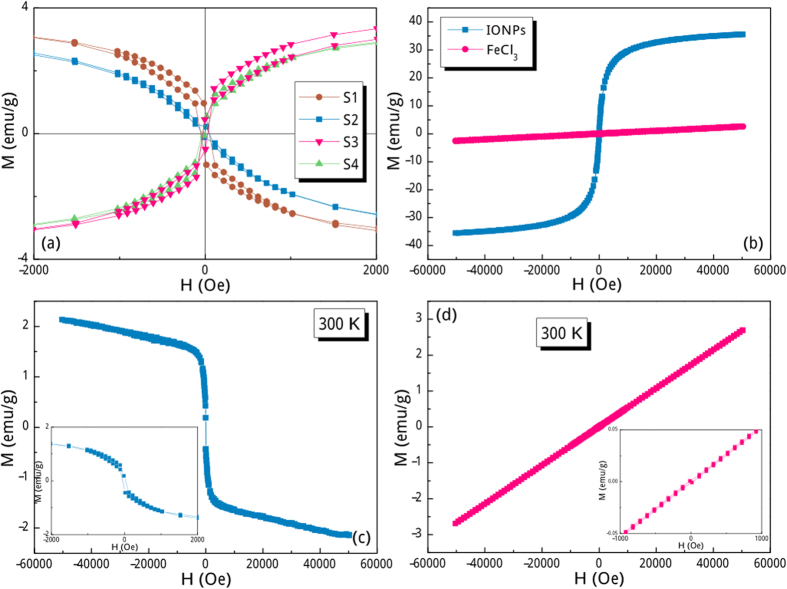
(**a**) Low-field hysteresis loops of IONPs@Fe^3+^ with different zeta potentials measured at 300 K. Room-temperature hysteresis loops of (**b**) bare IONPs and FeCl_3_ reference sample, (**c**) IONPs (ca. 5 nm)@Fe^3+^, and (**d**) IONPs (ca. 2 nm)@Fe^3^. The low-field hysteresis loops are shown as insets.

The room-temperature magnetic hysteresis loops are shown in Fig. 3, in which the saturation parts for S1 and S2 locate in the 2^nd^ and 4^th^ quadrants being quite different from the cases of S3 and S4. That is to say, the magnetization direction of S1 and S2 is totally opposite to the direction of the applied field both in the descending and ascending branches of hysteresis loops. These are the conclusive proof of SRMH in iron oxides because all these four particulate samples were correctly positioned at the detection coil saddle point before the SQUID magnetometer measurements were performed. Close inspection of Fig. 3 confirms that the occurrence of SRMH phenomenon seemingly depends on the zeta potential values of the sample. The degradation of SRMH as decreasing zeta potential values starts from the high-field magnetization, which exhibits non-overlapped and discontinuous magnetization hops of both descending and ascending branches (Fig. 3b). These erratic hops are not just artifacts and are reproducible, to some extent, by measuring the hysteresis loop of S2 in another SQUID device (Supplementary Figure S1). It can be confirmed that these erratic jumps will certainly happen in S2 even though its corresponding magnetic field value follows no rule. Further decreasing the zeta potential value will yield normal hysteresis loop but still with non-overlapped descending and ascending branches in the positive high-field range, as shown in Fig. 3c. These two branches will totally superpose when the zeta potential value is very low (Fig. 3d). Interestingly enough, the coercive fields of these samples are roughly below 50 Oe (Fig. 4a) and their saturation behaviors (Fig. 3) are akin to the superparamagnetism of bare IONPs (Fig. 4b). Based on these scenarios, the observed unique SRMH phenomenon cannot be solely classified as diamagnetism because of its saturation character, whereas it also cannot be solely classified as weak ferromagnetism or superparamagnetism due to its diamagnetic feature. The saturation magnetization (*M*_S_) of bare IONPs is ca. 35.6 emu/g (Fig. 4b), which is significantly lower than the bulk value for magnetite and maghemite (~82–93 emu/g^12^,^13^). This decrease in *M*_S_ can be ascribed to the effect of increased thermal fluctuation near the surface of IONPs or that of the magnetically disordered surface formed as a result of the large surface-to-volume ratio associated with the fine particle size^14^,^15^,^16^,^17^. It is noteworthy that the absolute *M*_S_ values of four IONPs@Fe^3+^ samples are very close (ca. 4.6 emu/g, Fig. 3), but they are much lower than that of the bare IONPs sample. This significant *M*_S_ falloff can be largely attributed to the presence of surface Fe^3+^ shell, which exhibits typical paramagnetism (Fig. 4b) and acts as a magnetic inert layer. In this case, the weight percent of coated Fe^3+^ cations can be roughly estimated as 87.1% by completely neglecting their magnetic contribution. Considering the size of bare IONPs being of 8 nm, the Fe^3+^ shell thickness is calculated to be above 0.5 nm, which is in excellent consistency with the TEM result. Notably, owing to their different equilibrium adsorption capacity in different FeCl_3_ aqueous solutions, the amount of Fe^3+^ cations are quite disparate for these four IONPs@Fe^3+^ samples, but the difference of their Fe^3+^ shell thickness is not that apparent. Different amount of Fe^3+^ cations located on bare IONPs will not significantly increase the weight of IONPs due to the finite shell thickness, but it may has different arrangement around IONPs and greatly affect the magnetic couplings between the superparamagnetic core and paramagnetic shell. In this case, the absolute *M*_S_ values of these four IONPs@Fe^3+^ samples are very close, but their magnetization behaviours are quite different.

The presence of paramagnetic Fe^3+^ shell and superparamagnetic IONPs core will contribute to the observed SRMH in IONPs@Fe^3+^. Along this line, two possible reasons are carefully considered. The first one is related to a demagnetizing field created by surface charge^18^. The actual field acting on the Fe^3+^ shell is the difference between the applied field and the demagnetizing field, and can be negative in a positive applied field. This explanation is also invalid for the observed SRMH in this study because the magnetization of IONPs core is much stronger than that induced by the surface demagnetizing field.

The second reason is related to a progressive magnetization process from the outside to the inside of IONPs@Fe^3+^. The directions of magnetic moments in IONPs@Fe^3+^ are randomly distributed under zero field due to strong thermal fluctuation. When an applied magnetic field penetrates these NPs, the magnetic moments in the Fe^3+^ shell firstly response to the external field and orient slightly toward the field direction to yield weak paramagnetism. Almost simultaneously, the magnetic moments in IONPs core are magnetized and negatively exchange-coupled with the Fe^3+^ moments in the vicinity of the core via superexchange interaction. The negative exchange coupling between IONPs core and Fe^3+^ shell originates from the spin-disordered feature of the shell, wherein the magnetic couplings are highly frustrated and hence leading to a severe degradation of host ferromagnetism. In this sense, this shell is akin to a magnetic dead layer of IONPs, which can negatively couple with the core^19^,^20^,^21^. Eventually, the directions of magnetic moments in superparamagnetic IONPs core are roughly antiparallel to the applied magnetic field. That is to say, the presence of pre-magnetized Fe^3+^ shell and its negatively magnetic exchange coupling with post-magnetized IONPs core is the root cause for the observed SRMH phenomena. Owing to the disordered moment distribution in paramagnetic Fe^3+^ shell, the negatively exchange-coupled magnetic moments in superparamagnetic IONPs core is of prominent disordered feature, which contributes to the decreased saturation magnetization (ca. 4.6 emu/g, Fig. 3) to a certain extent. It should be noted that the more magnetic moments involved in the negative exchange coupling in the core-shell region, the more obvious SRMH phenomenon can be observed. Along this line, the Fe^3+^ cation-disordered shell should be sufficiently thick or the IONPs core should be appropriately small. To verify this point, the Fe^3+^ concentration in FeCl_3_ aqueous solution was intentionally adjusted to 5, 2, 1 and 0.5 mol/L, and the resultant S1 and S2 samples exhibit obvious SRMH as expected, whereas S3 and S4 samples show normal magnetic behaviors (Fig. 3). On the other hand, if the size of IONPs core is further decreased, the number of its surface magnetic moments will greatly increase and they can strongly couple with the Fe^3+^ shell to yield well pronounced SRMH. Figure 4c shows that IONPs (ca. 5 nm)@Fe^3+^ are of typical SRMH feature even though they are synthesized by soaking in a lower concentration of FeCl_3_ aqueous solution (1 mol/L). Most notably, if the IONPs core is too small, almost all the magnetic moments locate at its surface, therefore the whole IONPs@Fe^3+^ may well not exhibit SRMH behavior due to dearth of enough superparamagnetic moments in the core. Figure 4d confirms that the IONPs (ca. 2 nm)@Fe^3+^ exhibit apparently paramagnetic behaviors rather than SRMH even though they are synthesized by soaking in a FeCl_3_ aqueous solution of 5 mol/L. More specifically, the *M*_S_ falloff from 35.6 to 4.6 emu/g indicates that the macrospins of the core may be not significantly above the total moments of the surface Fe^3+^ cations. To validate this point, we estimate the ratio of surface cation volume to the whole volume in bare IONPs. Assuming the average diameter of iron cations to be 0.6 nm, the surface cation volume accounts for 38.6%, 56.1%, 93.6% of the total volume of IONPs with diameters of 8 nm, 5 nm, 2 nm, respectively. This means a considerable amount of iron cations in bare IONPs locate at the surface and will contribute to the negatively exchange coupling to the adsorbed Fe^3+^ cations in FeCl_3_ aqueous solutions. An extreme case occurs in 2 nm-sized IONPs, in which nearly 94% cations locate at the surface, and magnetically couple with the adsorbed Fe^3+^ cations. In this case, the macrospins of remanent ~6% cations in the core are unable to exhibit their inherent superparamagnetism. Therefore the paramagnetic shell dominates the magnetic behaviour of IONPs (ca. 2 nm)@Fe^3+^ (Fig. 4d).

Collectively, the observed SRMH behavior in IONPs@Fe^3+^ can be easily modulated by tailoring IONPs size and altering Fe^3+^ concentration in aqueous solution. The latter approach is extraordinarily facile and versatile for the synthesis of a high yield of man-made materials with tunable SRMH features.

## Conclusions

In summary, a facile and versatile approach to scalable synthesis of SRMH iron oxides was proposed in this report. The feasibility of this approach depends exclusively on the zeta potential values of raw iron oxides. The negative enough zeta potentials are favorable for these iron oxides to electrostatically absorb Fe^3+^ cations to form a paramagnetic shell. Under an applied magnetic field, this shell will be firstly magnetized and its negative coupling with the post-magnetized iron-oxide core will result in unique SRMH behaviors in these materials. The observed SRMH phenomena can be modulated by changing the core size or by simply controlling Fe^3+^ concentration in aqueous solution.

## Methods

All chemicals were analytical grade and used as received without further purification. In a typical experimental procedure, 2 mmol of FeCl_3_·6H_2_O was dissolved into 50 mL of deionized water. Then, 10 mL of Na_2_CO_3_ aqueous solution (0.6 mol/L) was added with vigorous stirring. Five minutes later, 0.5 g of ascorbic acid was added into the above solution and stirred for another ten minutes. Finally, the mixture was transferred into a Teflon-lined stainless-steel autoclave with the capacity of 100 mL for hydrothermal treatment at 200 °C for 3 h. After the autoclave had cooled down to room temperature naturally, the precipitate was separated by centrifugation, washed successively with distilled water and absolute ethanol, and dried in the air at 60 °C for 4 h. Then the as-prepared IONPs were separately soaked into four sets of FeCl_3_ aqueous solutions with Fe^3+^ concentration of 5, 2, 1 and 0.5 mol/L for 1 h. The finally obtained IONPs@Fe^3+^ were separated by centrifugation, washed successively with distilled water and absolute ethanol, and dried in the air at 60 °C for 4 h.

The XRD patterns were recorded on a powder X-ray diffractometer (Rigaku D/max-rA) equipped with a rotating anode and a Cu K_α1_ radiation source (λ = 1.5406 Å) at a step width of 0.02°. The Raman spectrum was recorded using a Super LabRam microscopic Raman spectrometer (Labram, Jobin Yvon, France, a He-Ne laser with an excitation wavelength of 532 nm). X-ray photoelectron spectroscopy (XPS) was performed on VG ESCALAB 220i-XL system equipped with a monochromatic X-ray source in an ultra-high-vacuum chamber at a pressure lower than 1.0 × 10^−9^ Torr. Peak positions were referenced to the adventitious C 1 s peak taken to be 284.8 eV. Scanning electron microscope (SEM) images were collected on a field -emission scanning electron microscope (JEOL JSM-6700F). Transmission electron microscopy (TEM) was performed on the JEM-2100 TEM with an operating voltage of 200 kV. Dynamic light scattering (DLS, Zetasizer Nano, Malvern Instrument) was used to determine zeta potential values of the samples. The amount of Fe^3+^ cations adsorbed on IONPs was determined by inductively coupled plasma-optical emission spectrometer (ICP-OES, Perkin-Elmer, Optima 8000).

The magnetic measurements of powder samples were conducted by superconducting quantum interference device (SQUID) magnetometry (MPMS-XL5, MPMS-XL7, Quantum Design). The test accuracies were beyond 0.9 and 0.99 for the low-field (around the origin) and high-field range in the hysteresis loops, respectively. The unique SRMH phenomena discussed in this work is not related to SQUID settings and sample position. It is reported that if the sample is set inappropriately the voltage becomes linear function of both *M*_x_ and *M*_z_^1^. Here *M*_x_ and *M*_z_ are parallel and normal components of magnetization vector, respectively. On condition that the hysteresis loop is measured in a magnetic field applied along the hard axis, *M*_z_ in a low field range can be much larger than *M*_x_ and the contribution of *M*_z_ becomes so large that an inverted hysteresis of the induced voltage *vs.* applied field may be observed for this asymmetric sample settings. However, this explanation is not valid for the observed SRMH phenomena at least in two aspects: (i) all of the inverted hysteresis loops were reported in films^18^,^22^,^23^,^24^,^25^ and measured in the field direction along the hard axis, which is a common necessary condition required to observe suchlike experimental artifacts. The IONPs@Fe^3+^ in our experiment is particulate and their hard axes are disorderly distributed. It is highly improbable to apply a magnetic field along or around the hard axes of all NPs to yield experimental artifacts due to incorrect sample position; (ii) the phenomenon of inverted hysteresis loops in this explanation differs significantly from SRMH in this study. The former one is only observed in a low field range, during which *M*_z_ should be apparently larger than *M*_x_, whereas the latter one occurs in the whole range of the applied magnetic field.

## Additional Information

**How to cite this article**: Ma, J. and Chen, K. Modulated self-reversed magnetic hysteresis in iron oxides. *Sci. Rep.* **7**, 42312; doi: 10.1038/srep42312 (2017).

**Publisher's note:** Springer Nature remains neutral with regard to jurisdictional claims in published maps and institutional affiliations.

## Supplementary Material

Supplementary Information

## Figures and Tables

**Table 1:** Summaries of parameters for four IONPs@Fe^3+^ samples. The deviations are shown in parentheses.

Samples	Fe^3+^ concentrationmol L-1	Zeta potentialmV	Adsorbed Fe^3+^ cationsmg g-1
S1	5	19.3 (±3.6)	66 (±4.2)
S2	2	15.8 (±2.3)	62 (±3.7)
S3	1	2.7 (±2.6)	59 (±4.1)
S4	0.5	−9.2 (±3.2)	55 (±5.0)
