# Supplementary material for: Modulated self-reversed magnetic hysteresis in iron oxides
Source: Sci Rep. 2017 Feb 21;7:42312. doi: 10.1038/srep42312 (PMC5318991; doi:10.1038/srep42312)
Supplement: Supplementary Information [file srep42312-s1.pdf]

Supplementary Information

Modulated self-reversed magnetic hysteresis in iron oxides

*Ji Ma and Kezheng Chen**

Lab of Functional and Biomedical Nanomaterials, College of

Materials Science and Engineering, Qingdao University of

Science and Technology, Qingdao 266042, China.

* To whom correspondence should be addressed. Tel: +86-532-84022509. Fax: +86-532-84022509. E-Mail: kchen@qust.edu.cn

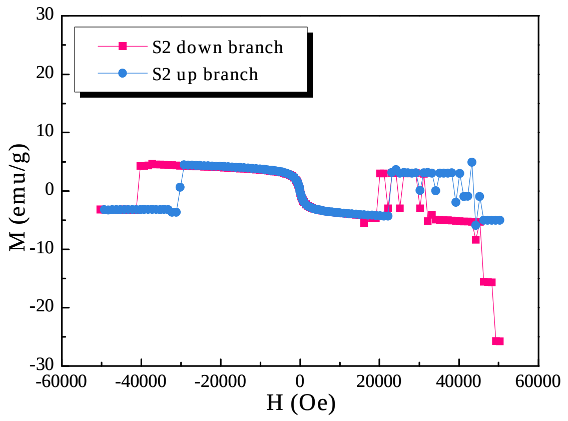

Figure S1 Room-temperature magnetic hysteresis loop of S2 measured in another SQUID device (MPMS-XL7).
